# The protein kinase CK2 catalytic domain from *Plasmodium falciparum*: crystal structure, tyrosine kinase activity and inhibition

**DOI:** 10.1038/s41598-018-25738-5

**Published:** 2018-05-09

**Authors:** David Ruiz-Carrillo, Jianqing Lin, Abbas El Sahili, Meng Wei, Siu Kwan Sze, Peter C. F. Cheung, Christian Doerig, Julien Lescar

**Affiliations:** 1 School of Biological Sciences, 60 Nanyang Drive, Nanyang Technological University, Singapore, 637551 Singapore; 2 Department of Biological Sciences Xi’an Jiaotong-Liverpool University111 Ren’ai Road, Dushu Lake Higher Education Town, Suzhou, 215123 People’s Republic of China; 3 Department of Microbiology, Monash University, Clayton, Australia; 4 Nanyang Institute of Structural Biology, Experimental Medicine Building, 59 Nanyang Drive, Singapore, 636921 Singapore

## Abstract

Malaria causes every year over half-a-million deaths. The emergence of parasites resistant to available treatments makes the identification of new targets and their inhibitors an urgent task for the development of novel anti-malaria drugs. Protein kinase CK2 is an evolutionary-conserved eukaryotic serine/threonine protein kinase that in *Plasmodium falciparum* (*Pf*CK2) has been characterized as a promising target for chemotherapeutic intervention against malaria. Here we report a crystallographic structure of the catalytic domain of *Pf*CK2α (D179S inactive single mutant) in complex with ATP at a resolution of 3.0 Å. Compared to the human enzyme, the structure reveals a subtly altered ATP binding pocket comprising five substitutions in the vicinity of the adenine base, that together with potential allosteric sites, could be exploited to design novel inhibitors specifically targeting the *Plasmodium* enzyme. We provide evidence for the dual autophosphorylation of residues Thr^63^ and Tyr^30^ of *Pf*CK2. We also show that CX4945, a human CK2 inhibitor in clinical trials against solid tumor cancers, is effective against *Pf*CK2 with an IC_50_ of 13.2 nM.

## Introduction

Two hundred million cases of malaria occur annually resulting in half-a-million deaths of which most are children from sub-saharian Africa^1^. Malaria is caused by infection with protozoan parasites of the genus *Plasmodium* with *Plasmodium falciparum* and *Plasmodium vivax* accountable for the most virulent forms of the disease. The emergence of parasite resistance to most of the available antimalarial treatments, including artemisinins, has already been observed, making the discovery of alternative therapeutic targets a pressing need^1^. Eukaryotic protein kinases (ePKs) form a large family of enzymes with major roles in the regulation of many cellular processes^2^. The therapeutic relevance and druggability of ePKs is emphasized by the growing number of kinase inhibitors that are used in the clinic or have entered clinical trials to treat various conditions such as cancer^3^.

Not surprisingly, protein kinases are also pivotal to regulate cellular processes in pathogenic parasites such as *Leishmania*^4^ or *Plasmodium*^5–7^. The genome of *Plasmodium falciparum* includes more than 80 putative protein kinases^8^, several of which have been shown to be indispensable for the parasite survival^5,9,10^. The relevance of the *Plasmodium* kinome in the intrincated life cycle of the parasite, is apparent from the fact that 50% of its proteome becomes phosphorylated during the erythrocytic stage, with a vast majority of proteins displaying multiple phosphorylation sites^11^. In this context, a central regulatory role has been suggested for the *Plasmodium falciparum* protein kinase CK2 (*Pf*CK2)^11^, as an enzyme regulating the activity of several other important kinases. Moreover, reverse genetics experiments showed that the catalytic α subunit α of *Pf*CK2 is essential for pathogen survival, during its replicative cycle whithin the host red blood cells^5,6^, making of *Pf*CK2 a promising drug target. In contrast to many kinases that only become activated upon receiving specific regulatory signals, CK2 is a pleiotropic and constitutively active Ser/Thr kinase that phosphorylates and regulates the activity of a suprisingly large number of proteins, including transcriptional activators^12^. Unlike the human enzyme (hCK2), *Pf*CK2 undergoes autophophorylation at Thr^63^ but not at Tyr^186^, a residue located within its activation loop^13^. Here, using mass spectrometric analysis, we show that *Pf*CK2 also displays Tyrosine kinase activity, leading to autophosphorylation at Tyr^30^. The human hCK2 ortholog is built up of either homo- or hetero-tetramers formed by combinations of two catalytic (α and α′) subunits and two regulatory (β) subunits^14^. In contrast, *Pf*CK2 achieves its quaternary structure using one single catalytic (α) and two regulatory (β_1_ and β_2_) subunits. The cytoplasmic and nuclear localization of *Pf*CK2 are consistent with pull-down experiments linking *Pf*CK2 with the regulation of nucleosome assembly, suggesting a role for this kinase during mitotic cell division^6^. The three-dimensional structure of hCK2 has already been well characterized in its free form, in complex with the non-hydrolysable ATP analogue AMPPNP, and in complex with several potent small molecule inhibitors targeting the enzyme ATP binding site^14–18^. However an experimental *Pf*CK2 structure is still unavailable. The 65% level of amino acid sequence identity between the catalytic *Pf*CK2 and hCK2 α subunits, indicates that both enzymes share a well conserved overall structure. However, determination of the *Plasmodium* enzyme 3D structure would allow a precise comparative structural analysis with its human ortholog that could lead to the identification of enzyme regions, including at its ATP binding site, amenable to specific inhibition. Here, as a first step towards this goal, we report the bacterial expression, purification and crystallization of an enzymatically inactive mutant of *Pf*CK2α having the mutation D179S and its crystal structure bound to ATP at 3.0 Å resolution. Using the wild-type protein expressed in the same conditions, we provide experimental evidence from mass spectrometry of a dual Serine/Threonine and Tyrosine kinase activity of *Pf*CK2. Using a peptide substrate, we also study the impact of single and double mutations (to Ala or Asp) targeting phosphorylable residues Tyr^30^ or Thr^63^ on *Pf*CK2 kinase activity. Finally, we show that CX4945, a potent hCK2 inhibitor^18^, inhibits PfCK2 with an IC50 of 13.2 nM.

## Results

### Expression and Purification of *Pf*CK2α and its mutants

**Figure 1:**
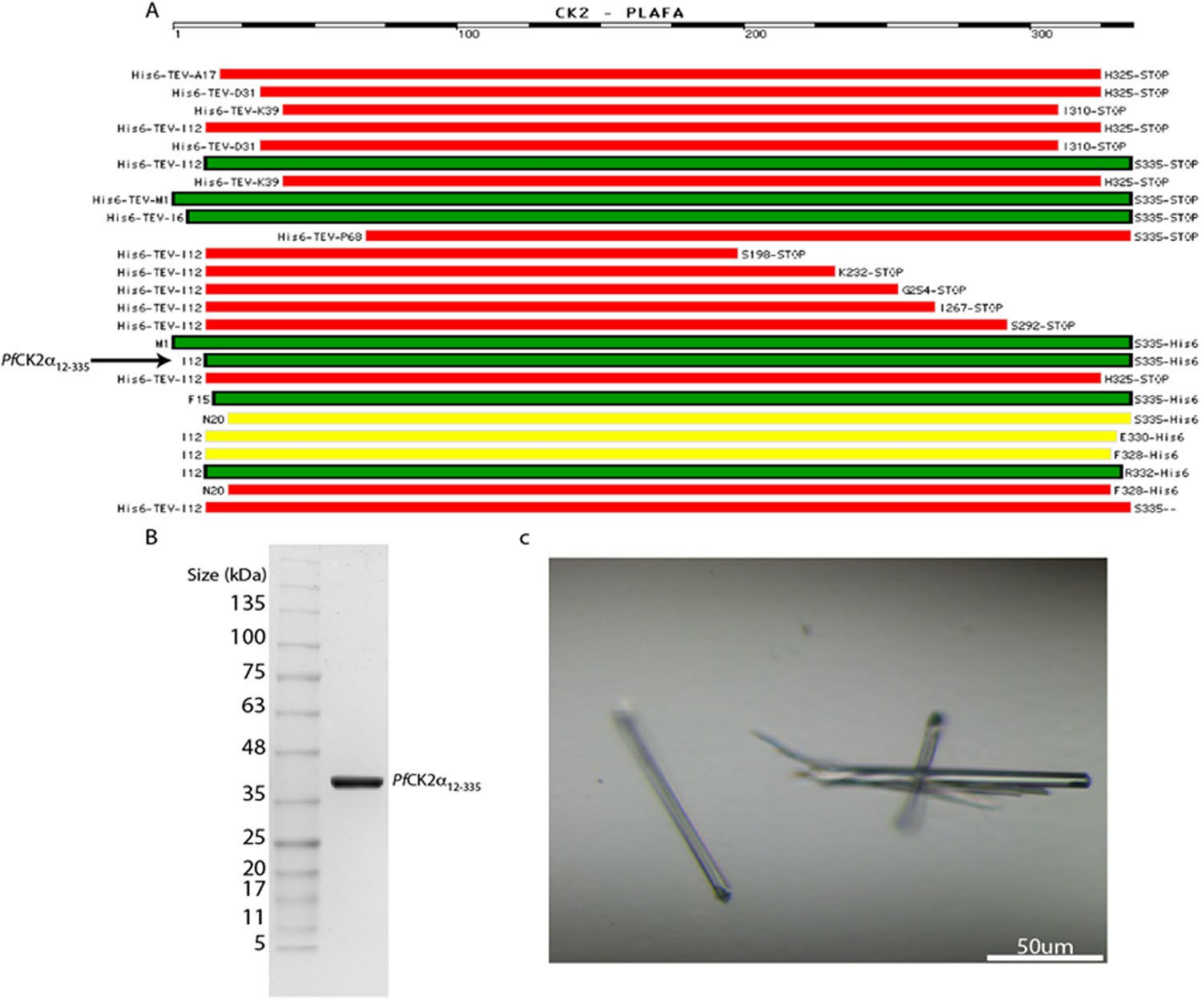
Protein Expression. (**A**) Selected constructs from the series generated for identifying the protein boundaries yielding the most soluble and stable *Pf*CK2α enzyme when expressed in *E. coli*. Red, yellow and green rectangles represent insoluble, marginally soluble, and soluble constructs respectively. The position of the hexahistidine tag is indicated as “His6” and theTEV cleavage sites, and amino- and carboxy terminal residues labelled. (**B**) 12% SDS-PAGE analysis of the purified *Pf*CK2α constructs (wild-type and single mutant) used in this study spanning residues Ile^12^-Ser^335^. (**C**) Needle-shaped *Pf*CK2α_D179S_ crystals obtained after optimization using micro-seeding (see methods).

We used a multi-construct approach in *E. coli* in order to obtain protein yields suitable for X-ray crystallography. We screened more than 40 protein constructs having either N- or C-terminal truncations with the tag located at the N- or C-terminal end of the protein^19^. We found that N-terminal truncations of *Pf*CK2α usually yielded insoluble proteins, while C-terminal truncations improved protein solubility and stability (Fig. 1A). Moreover, we observed a significant improvement in protein stability when the hexa-histidine tag used for affinity purification was displaced from the N- to the C-terminus of the protein. A protein fragment spanning residues Ile^12^ to Ser^335^ of *Pf*CK2α was used for large-scale expression, purification and enzymatic activity measurement. For crystallization and structure determination, the “Aspartate 179 to Serine” single mutant of the same fragment (*Pf*CK2α_D179S_) was designed (Fig. 1B). Non-phosphorylatable mutants, namely Thr^63^ to Alanine (*Pf*CK2α_T63A_) and Tyr^30^ to Alanine (*Pf*CK2α_Y30A_), as well as double mutant *Pf*CK2α_T63AY30A_, were generated to examine the effect of autophosphorylation at Thr^63^ and Tyr^30^ on kinase activity. Likewise, phospho-mimetic mutants, namely Thr^63^ to Aspartate (*Pf*CK2α_T63D_) and Tyr^30^ to Aspartate (*Pf*CK2α_Y30D_) as well as double mutant *Pf*CK2α_T63DY30D_, were also obtained.

### PfCK2α is an active Ser/Thr kinase

**Figure 2:**
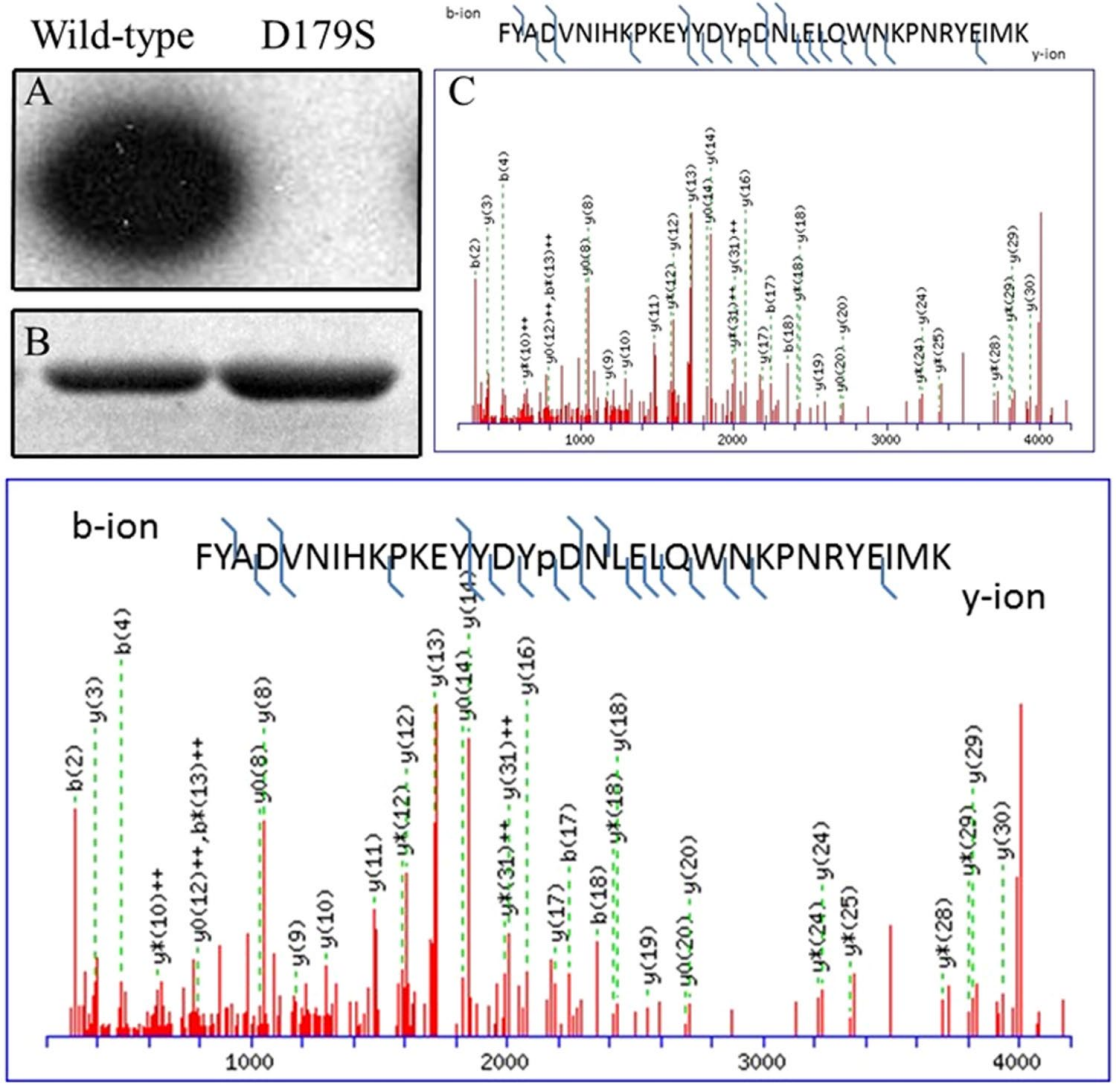
Autophosphorylation activity of *Pf*CK2. (**A**) Autoradiograph kinase activity assay was carried out using 2 μg of wild type (lane 1) or D179S mutant *Pf*CK2 (lane 2) with Mg [γ^32^P]-ATP. (**B**) The proteins were resolved by SDS-PAGE, stained with Coomassie blue and their activity analysed by autoradiography. (**C**) Annotated MS/MS spectra showing identification of Y30 phosphorylation using the Mascot protein database search software. The Tyr^30^ (labeled Y16 in the spectrum) is unambiguously identified as it is sandwiched by a series of y-ions [y16,y17,y18(phosphorylation site),y19,y20]. y13 ion was trimmed 10 times to show the weaker ions in the spectrum.

The activity of the protein was assessed using [γ^32^P]-ATP based radiograms (see methods). To unequivocally ascertain phosphorylation to intrinsic *Pf*CK2α activity, an inactive kinase *Pf*CK2α_D179S_ single mutant was used as negative control (the expression of an alanine mutant at position 179 proved to be insoluble). The “179-DWG-181” motif in the catalytic loop is an absolutely conserved amino acid sequence motif responsible for ATP and Mg^2+^ binding by the kinase and whose mutation typically causes *bona fide* loss of kinase activity. The results of an enzymatic assay displayed in Fig. 2A,B unequivocally demonstrate that the wild-type *Pf*CK2α enzyme is catalytically active and confirmed *Pf*CK2α autophosphorylation activity.

### Identification of autophosphorylation sites

**Figure 3:**
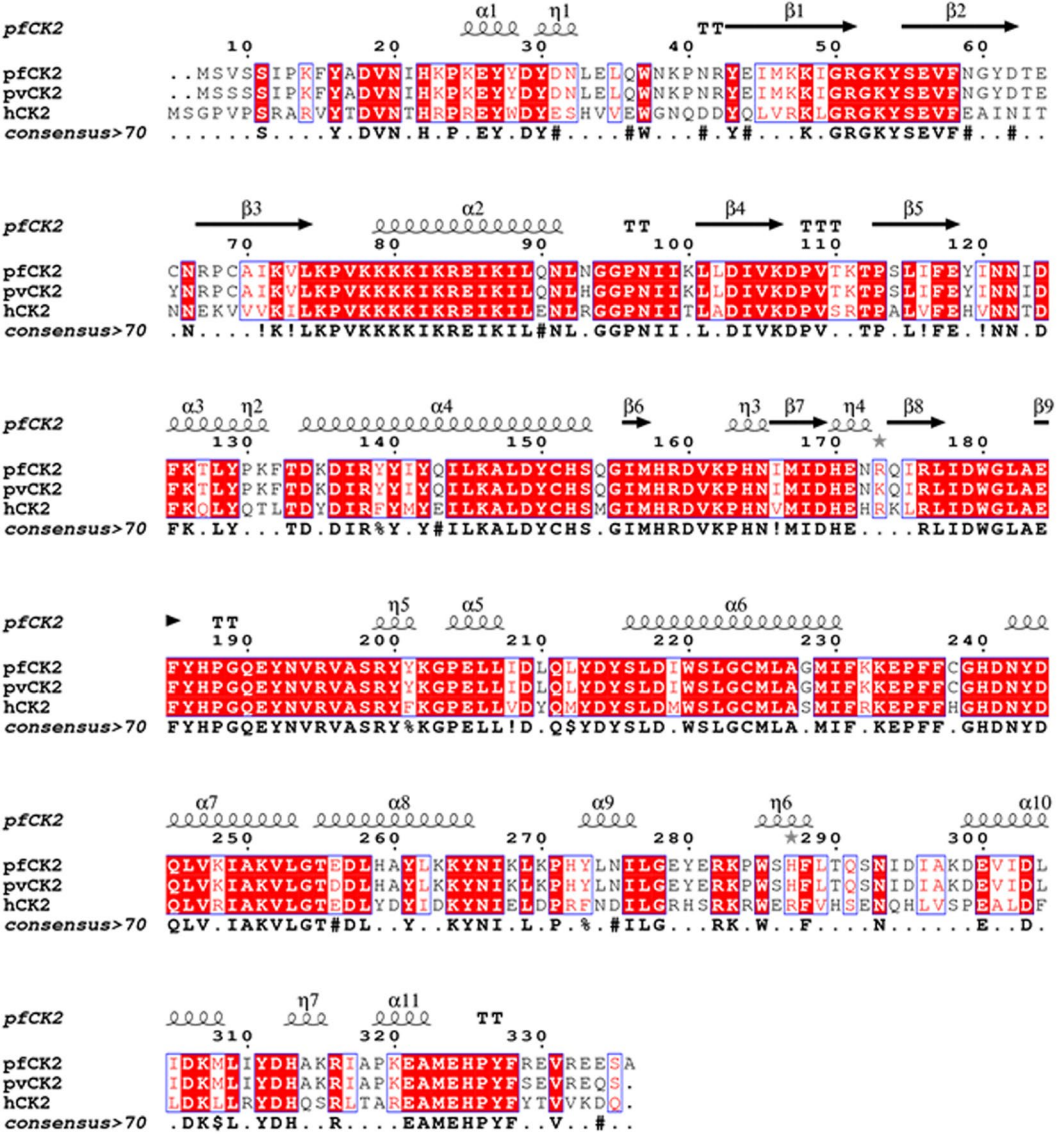
Structure-based sequence alignment of CK2 sequences from two species of the *Plasmodium* parasite and of human CK2. Amino-acid sequence alignment of alpha subunits of CK2 proteins from human **(**P68400), *Plasmodium vivax* **(**A0A0J9V3K6) and *Plasmodium falciparum* **(**Q8IIR9). The figure was prepared using http://espript.ibcp.fr^45^. The secondary structures elements for *Pf*CK2 (this work) are displayed above the alignment, with the helices depicted as springs and strands as arrows. The hCK2 C-terminal tail (ARMGSSSMPGGSTPVSSANMMSGISSVPTPSPLGPLAGSPVIAAANPLGMPVPAAAGAQQ) was removed from the alignment because no equivalent residue is present in *Pf*CK2α.

Mass spectrometry (MS) analysis of the active *Pf*CK2α identified four autophosphorylation sites: two presenting as major at Tyr^30^ and Thr^63^, and two as minor phosphorylation sites at Tyr^61^ and Ser^55^ (Fig. 2C and Table 1), representing approximately 90% and 10% of the identified phosphorylated peptides respectively. Moreover, the same MS analysis conducted using the catalytically inactive *Pf*CK2α_D179S_ protein proved that the post-translational modifications observed were indeed the result of catalytic autophosphorylation and were not caused by *E. coli* enzymes **(**Fig. 2A**)**. The identified phosphorylation sites agree with the acidic nature of the CK2 peptide substrates recognition sites^12^, and confirm the catalytic specificity that was previously attributed to *Pf*CK2^13^. We could corroborate Thr^63^ as one of the two major phosphorylation sites, together with Tyr^30^ that unexpectedly also appeared to undergo phosphorylation (Fig. 2C and supplementary data). This dual autophosphorylation pattern of *Pf*CK2 differs from the single phosphorylation pattern described previously^13,20^, but is in agreement with the Tyrosine specificity found in the autophosphorylation pattern of the human enzyme^13,20^. Unlike Tyr^186^ (equivalent to Tyr^182^ from hCK2 in the kinase activation loop)^13^, Tyr^30^ does not belong to a conserved autophosphorylation site. A structure-based alignement of the amino-acid sequces of hCK2, *Pf*CK2 and *Pv*CK2 is presented in Fig. 3. In the 3D structure, the hydroxyl moiety of Tyr^30^ is within hydrogen bond distance from Glu^184^ and in the vicinity of the putative peptide substrate binding site delinated by the basic patch encompassing residues Lys^78^-Arg^84^. The functional role of these two autophosphorylation sites was explored by site-directed mutagenesis followed by kinase activity (see below).

**Table 1:** Phosphorylation sites (bold and underlined) identified in the recombinant *Pf*CK2α derived from this work.

Major phosphorylation sites	25-KEYYD**Y**DNLEL-35
	52-GKYSEVFNGYD**T**ECNRP-68
Minor phosphorylation sites	58-FNG**Y**DTECN-66
	52-GKY**S**EVFNGYD-62

### Catalytic activity measurements

To explore the functional relevance of the autophosphorylation sites, the enzymatic activity of Tyr^30^ and Thr^63^ *Pf*CK2 mutants were assayed in a peptide-based phosphorylation assay (Table 2). Wild type and mutant D179S defined the catalytic range of *Pf*Ck2 with respectively 1498.74 ± 43.22 and 1.48 ± 3.32 units of activity (ua) per mg of purified protein. Single mutants Tyr^30^Ala and Thr^63^Ala had reduced activity of 877.52 and 360.68 ua/mg, respectively, demonstrating a direct impact of autophosphorylation in regulating *Pf*CK2 kinase activity. Taking into account that these measurements were made only with the catalytic α subunit, these changes indicate that both Tyr^30^ and Thr^63^ play a direct role in activation that might be further modulated in the presence of the regulatory subunits. *Pf*CK2 proteins having single mutation of Tyr^30^ or Thr^63^ to aspartate had further reductions in catalytic activities compared to the corresponding Ala mutants (Table 2). Moreover, *Pf*CK2 having double Tyr^30^ and Thr^63^ Aspartate mutant had negligible kinase activity. This possibly suggests that sequential autophosphorylation is needed for activation of *Pf*CK2., an event that could not be mimicked by the introduction of a shorter carboxylic side chain as presented in the form of an Asp residue.

**Table 2:** PfCK2α variants activity measurements using the peptide [RRRDDDSDDD] as phosphate acceptor for the incorporation of radiolabelled [γ^32^P]-ATP.

	Activity (Units/mg)
hCK2α	1385.86 ± 42.37
Wild type PfCK2α	1498.74 ± 43.22
PfCK2α_D179S_	1.48 ± 3.32
PfCK2α_T63A_	360.68 ± 36.13
PfCK2α_Y30A_	877.52 ± 151.14
PfCK2α_T63AY30A_	180.27 ± 3.27
PfCK2α_T63D_	202.42 ± 46.13
PfCK2α_Y30D_	649.77 ± 61.03
PfCK2α_T63DY30D_	6.71 ± 1.63

### Enzyme inhibition

**Figure 4:**
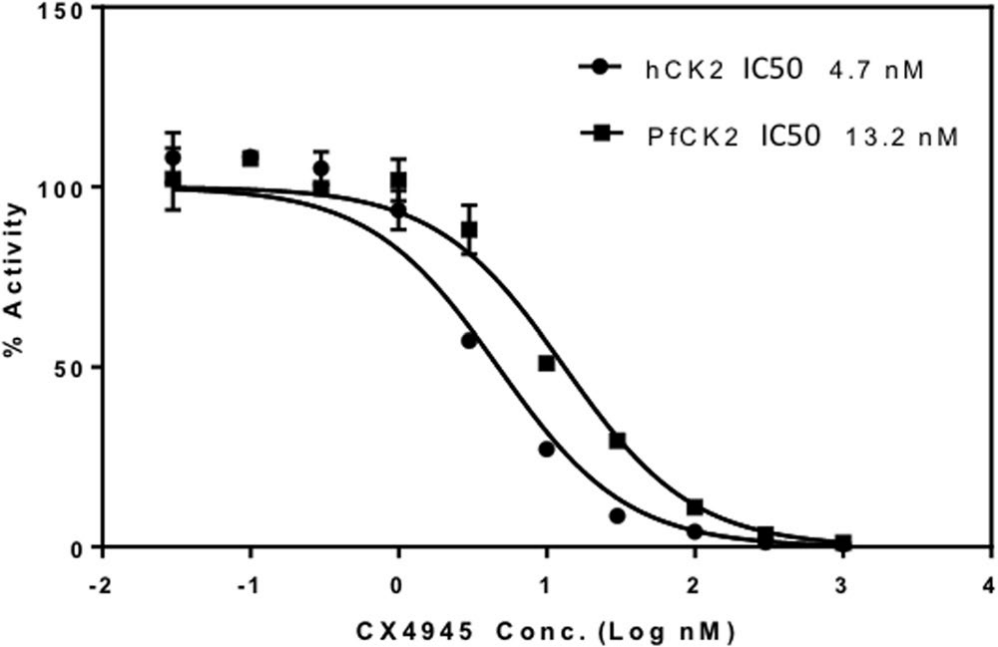
Effect of compound CX4945 on CK2 activity. The compound CX4945 displays concentration-dependent inhibition on both CK2 proteins used here, human (circles, hCK2) and *Plasmodium falciparum* (squares, PfCK2). The IC50 value of CX4945 for each protein is indicated. Data were analysed using the GraphPad Prism software using a Log [inhibitor] vs normalized response fit.

The human CK2 protein has been thoroughly characterized as a potential target for therapeutical treatment against cancer^21,22^. To explore whether CX4945 an ATP competitive inhibitor targeting hCK2 can be repurposed to target *Pf*CK2 for the treatment of malaria, we measured its IC50 for both enzymes. Our measurements show that both proteins can be inhibited (Fig. 4) with a three fold difference in IC_50_ value: Our result for hCK2 (IC50 of 4.7 nM) is in agreement with previous measurements of the CX4945 potency (1 nM IC_50_)^23^, while we find that CX4945 inhibits *Pf*CK2 with an IC50 value of 13.2 nM. The difference in IC50 between the two homologous enzymes, although small, indicates that their ATP binding sites are slightly different, which is consistent with the crystallographic structure determination reported below. Whether these structural differences could be exploited to modify CX4945 to specifically inhibit the parasite enzyme requires further medicinal chemistry studies informed by the present structural information. Interestingly, similar inhibitory responses were characterized in the case of quinalizarin^13^, suggesting that inhibitors bind the human and the *Plasmodium* proteins slightly differently, despite the close similarity of their active sites.

### Crystal structure determination

The wild type *Pf*CK2 was subjected to extensive crystallization trials. Initially, only severely twinned needle shaped crystals giving diffraction at best to about 3.4 Å were obtained with the wild type enzyme, and the resulting electron density maps were of poor quality. A significant improvement in crystals quality was obtained when the inactive mutant *Pf*CK2α_D179S_ was used. The difference possibly reflects sample hereogeneity provoked by autophosphorylation when using the wild type enzyme. The structure of the complex between the catalytically inactive mutant *Pf*CK2α_D179S_ with ATP was prepared by cocrystallization, transferring crystals seeds initially grown in the presence of protein, ATP and Mg^2+^ into a solution devoid of Mg^2+^ (see methods). Data collection and refinement statistics for *Pf*CK2α_D179S_ bound to ATP are presented in Table 3. The structure was refined to R_work_ /R_free_ = 0.192/0.231 at a resolution of 3.0 Å. Clear electron density was visible for all three molecules (named A, B, C) present in the asymmetric unit (Fig. S1A), allowing unequivocal assignement for of all main chain and a majority of side chain atoms for the polypeptide chain up to residue 328. Residues 329–335 at the C-terminal end of the protein are disordered. The structures of all three monomers were tightly restrained during refinement, resulting in r.m.s. deviations values for their main chain atoms between 0.21 Å and 0.24 Å after superposition (for a total of 316 α-carbon atoms)^24^. A structure-based alignement of the sequences of *Pf*CK2, *Pv*CK2 and hCK2 is presented in Fig. 3.

**Table 3:** Data collection and structure refinement statistics.

Source	PXIII beamline from Swiss Light Source
Wavelength (Å)	1.0
Space group	P2_1_2_1_2_1_
Unit cell (Å)	88.41/123.55/125.65 90/90/90
Molecules in the asymmetric unit	3
Resolution range (Å)	30–3.0 (3.17–2.99)
No. of observed reflections	124984 (18377)
No. of unique reflections	28155 (4252)
Completness	98.8 (94.7)
Multiplicity	4.4 (4.3)
Wilson B-factor (Å^2^)	66.51
(I/(σ)I)	7.82 (1.78)
^≠^R_merge_	19.8 (82.1)
CC_1/2_*	98.2 (61.4)
^‡^R_work_/R_free_	17.91/22.05
R.m.s.d bonds (Å)	0.008
R.m.s.d angles (°)	0.96
Overall B factor (Å^2^)	49.48
Protein/Water/ Ligand (ATP)	49.66/29.08/65.06
Ramachandran outliers(%)	0
PDB code	5XVU

^a^The numbers in parentheses refer to the last (highest) resolution shell.

^b^*R*_*merge*_ = Σ_h_Σ_i_|I_hi_ − <I_h_> |/Σ_h,i_ I_hi_, where I_hi_ is the *i*th observation of the reflection h, while <I_h_> is its mean intensity. Abbreviation a.u.: asymmetric unit. *CC1/2 = percentage of correlation between intensities from random half‐dataset.

Analysis of crystal contacts reveals interfaces between the three monomers extending over an area of approximately 700 Å^2^. Residues brought in extensive intermolecular contact originate from the C-lobe of molecule C and the N terminal arm preceeding the N-Lobe of molecule A. Interestingly, some crystal contacts involve residues from the P-loop, and phosphorylation site around Thr^63^ whose structure might be affected by crystal packing forces.

**Figure 5:**
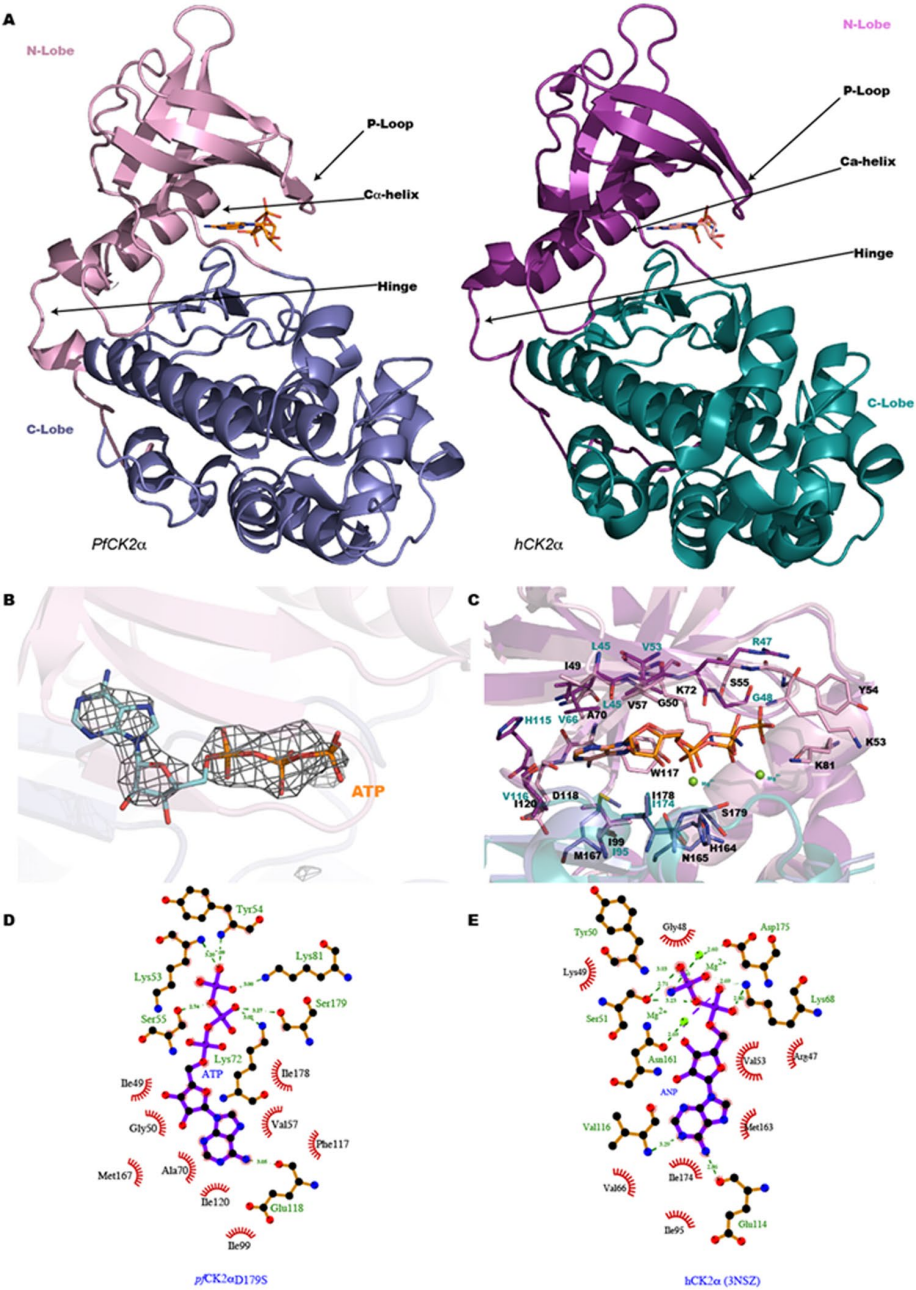
(**A**) Side-by side representation of the *Pf*Ck2α_D179S_ structure (left and hCK2 (right) shown in the same orientation. The N-terminal tail, the N-lobe and C lobe are displayed in different colors. Structural elements discussed in this paper, namely P-loop, hinge region and C-helix are indicated. The ATP (*Pf*CK2) and AMPPNP (hCK2) ligands bound in the active site are displayed as sticks. (**B**) Close up view of the bound ATP (sticks) in the *Pf*CK2 active site with an omit residual electron density map superimposed. (**C**) Superposition of how the substrate is bound by the PfCK2α_D179S_ and human enzyme: residues contributing to the active site of *Pf*Ck2α_D179S_ are displayed using the same color code as in panel A. The hCK2-AMPPNP structure (from PDB code: 2PVR) is shown in blue. ATP and AMPPNP substrates are shown as sticks and Mg ions as green spheres (from PDB code: 2PVR). Residues are labelled in black for *Pf*Ck2α_D179S_ and in blue for hCK2. (**D**,**E**) Flat representation of the atomic interactions (generated with Ligplot^45^) between PfCK2α_D179S_ and ATP (panel D, this work) and hCK2 (PDB code: 2PVR) and AMPPNP (panel E).

A residual difference electron density map at a level of 5 σ allowed unambiguous building of the bound ATP for each of the three molecules present in the asymmetric unit (Fig. 5). Here, the ATP moiety is bound in the active site in the absence of divalent metal ions (Mg^2+^) that are seen to coordinate the phosphate groups in nucleotide binding proteins, indicating that the energy contributions of active site residues are sufficient for substrate binding. Using isothermal titration microcalorimetry, we compared the binding parameters of *Pf*CK2α_D179S_ (the protein used for crystallization) for ATP in the presence and absence of Mg^2+^. These measurements returned a *K*_*D*_ value of 22.3 µM in the absence of Mg^2+^ and 16.3 µM in the presence of a concentration of 1 mM of Mg^2+^, confirming that the mutant protein can bind ATP in the absence of Mg^2+^ with the same 1:1 stoichiometry. The microcalorimetry data reveal a negative enthalpy change of ΔH indicating a binding mechanism involving polar interactions. This is in agreement with what is observed in the crystal structure of the complex. Consistent with the overall high level of amino-acid sequence identity of 65% (Fig. 3), *Pf*CK2α shares with its human ortholog the canonical kinase bi-lobular tertiary structure with a N-terminal domain arranged around a central β-sheet and a bundle of α-helices forming the C-terminal domain (Fig. 5A). The active site is located at the interface between both sub-domains forming a groove that penetrates deeply inside the protein (Fig. 5B). A structural comparison of *Pf*CK2α_D179S_ (monomer A) with hCK2 in its active (PDB codes 3NSZ and 2PVR)^18,25^ and inactive forms (PDB code 3FWQ)^26^ returns r.m.s.d. values of 0.85 Å, 1.22 Å and 1.56 Å (after superposing α-carbon atoms from residues 12 to 312), respectively.

### *Pf*CK2α_D179S_ adopts an active conformation similar to hCK2

We compared the structure of *Pf*CK2α_D179S_ α with both hCK2 and protein kinase A (PKA, PDB accession code 1ATP), the first member of the kinase family whose structure was thoroughly characterized^27^. PKA is frequently used for ascribing an active or inactive form to a novel kinase structure. Out of the eleven kinase motifs classified by Hanks and Hunter^28^, the following structural elements: αC-helix, hinge region and the P-loop are particularly relevant to define a kinase active or inactive conformation. These three motifs cooperate to build an active enzymatic form, with the P-loop, which is located between the first two antiparallel β-strands in the N lobe main β-sheet, showing the most significant conformational flexibility between various kinase structures^29^. Based on the conformations adopted by these three elements, the present crystal structure reveals that *Pf*CK2α_D179S_ most resembles an active conformation^26,30,31^. Superposition of *Pf*CK2α_D179S_ with hCK2 reveals that the N-lobe displays larger differences with r.m.s. deviations of respectively 0.81 (PDB code: 3NSZ), 1.41 (3WAR), 1.46 (2PVR) and 1.83 Å (3FWQ) for α-carbon atoms of residues 12–121. Interestingly, the RMSD in the C lobe are similar (~0.8 Å) regardless of the exact hCK2 structure used for comparison, over the 131–331 amino-acid range, indicating that the *Pf*CK2α_D179S_ structure displays a good degree agreement with all of them. The *Pf*CK2α_D179S_ structure displays a well aligned R-spine^32^, involving residues Leu^101^, Leu^89^, Trp^180^ and His^158^ and its C-spine, formed by residues Val^57^, Ala^70^, Phe^125^, Ile^166^, Met^167^ and Ile^168^, Met^225^ and Met^229^ are characteristic of an active state (Fig. S1B), despite the fact that Phe^125^ departs from the position occupied by Met^128^ in PKA^32,33^, and conforms more to its counterpart Phe^121^ in hCK2^25^. We also note that residues Ala^70^ and Val^57^ that are involved in the formation of the C-spine are not conserved compared to the residues found in hCK2: Val^53^ and Val^66^. Since their conformation is directly influenced by the adenine base upon substrate binding, this difference might be of relevance as small changes in the active site of CK2 have already been proven significant for determining the strength of inhibitor binding^34^.

Likewise, the hinge region encompassing strand β5 that links the N and C Lobes, including the polypeptide backbone between Phe^117^ and Phe^125^, adopts a conformation similar to the active forms of hCK2 (with RMSD for Cα atoms for residues 117–125 of 0.26 Å compared to the active hCK2 structure (3NSZ). Like in hCK2, the *Pf*CK2α_D179S_ hinge region accomodates the adenine moiety within the ATP binding site. Replacement of residue Val^116^ (hCK2) by Ile^120^ in *Pf*CK2α leads to a small alteration of the shape of the ATP binding pocket. This amino-acid substitution has an impact on ATP binding as the main chain amino group of Val^116^ (of hCK2) makes a direct contact with the N1 amine of the adenine base and allows the carbonyl group of Glu^114^ to form a hydrogen bond with the N6 of the adenine base. These contacts are absent in the *Pf*CK2-ATP complex (compare Fig. 5D,E)

The P-loop which includes residues Gly^49^–Tyr^54^ of *Pf*CK2α_D179S_ adopt an extended conformation similar to the one found in the active forms of CK2 (with RMSD 0.55 Å, 0.48 Å and 0.54 Å following superpositions with structures 3NSZ, 3WAR (the highest resolution structure available of an active CK2) and 2PVR respectively and it markedly differs from the inactive “collapsed” inactive form of CK2 (PDB code: 3FWQ), with a RMSD 2.35 Å. Nonetheless, several residues preceding the P-loop such as Met^46^ and Arg^51^, adopt a conformation distinct from either the active (RMSD 0.52 Å and 0.65 Å for structures 3NSZ and 2PVR respectively) or inactive hCK2 structures (RMSD 1.23 Å with PDB: 3FWQ). Interestingly, the β1 and β2 strands of *Pf*CK2α_D179S_ do not overlap with their counterpart structures in the active human structure (PDB code: 2PVR). Residue Gly^50^ is 3.28 Å away from the position adopted by the equivalent residue Gly^46^ in hCK2, which is due to a shift of the entire beta-sheet between the human and *Plasmodium* proteins Such displacements are induced by ATP binding (see below) and propagate across the beta blade with the Cα of residue Val^109^ of *Pf*CK2 displaced by 5.83 Å from the position adopted by Val^105^ in the active human enzyme.

### Protein-ATP interactions

**Figure 6:**
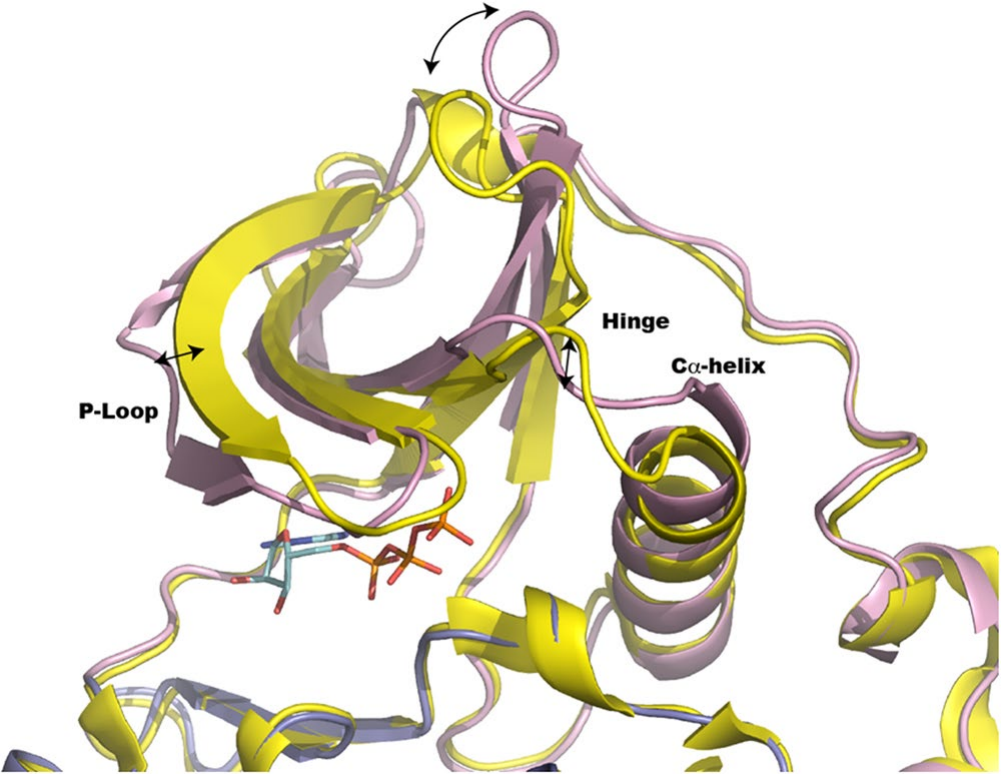
Conformational changes between the structure adopted by the N-Lobe of *Pf*CK2α_D179S_ (pink) and hCK2 (PDB code: 2PVR, yellow). A superposition of *Pf*CK2α_D179S_ (this work, colored in pink) and the active form of hCK2 (PDB code: 2PVR) shown in yellow. The three arrows show the main displacements described in the text at the level of the p-loop, Hinge and c-helix.

The atomic interactions between *Pf*CK2α_D179S_ and ATP are depicted in Fig. 5B,C. An analysis^35^ of the substrate-protein interface in *Pf*CK2α_D179S_ and hCK2 (PDB code: 2PVR) reveals that the *Plasmodium* protein contributes an interface area of 353 Å^2^ compared to 309 Å^2^ for hCK2 bound to an ATP analog. An important substitution, Asp^179^Ser, was engineered for the catalytic characterization of the enzyme that resulted actually in improved protein crystallizability. The loss of the catalytic activity due to the mutation of Asp^179^ appears to have drastic consequence not only in the catalytic activity of the protein (Fig. 2A) but remarkably also on the positioning of the phosphate groups of the ATP molecule compared to hCK2 (PDB code: 2PVR). However we note that this substitution has no effect on the conformation of the catalytic loop. The loss of the interaction between the α-phosphate and Asp^149^ results in the triphosphate moiety being buried more deeply in the *Pf*CK2 catalytic cleft, making ionic contact (distance of 3 Å) with Lys^81^ (Fig. 5D). As noted above, this closer contact formed with the “IGRG” motif (residues 49–52 of *Pf*CK2) by the ATP molecule displaces the β1 strand from the conformation it adopts in the active human enzyme. The main consequence of this conformational change is an active site whose hydrophobic character appears to be enhanced (Fig. 5C), with residues Gly^50^, Gly^52^, Val^57^, Ala^70^, Lys^72^, Ile^49^, Ile^120^ Glu^118^, Met^167^ and Ile^178^ participating in binding ATP (Fig. 5). Compared to hCK2, (Fig. 5B) several residues that bind ATP are conserved and superpose well in both structures (*Pf*CK2α_D179S_ and 2PVR), including Lys^72^ (using *Pf*CK2α numbering) which coordinates the α and β-phosphate groups of ATP and Glu^85^ that makes a salt bridge with Lys^72^. Besides the catalytically active residues, several other residues involved in ATP binding are also conserved, such as P-loop residues Gly^50^ to Tyr^54^ (Fig. 5A). However, as pointed above, in *Pf*CK2α_D179S_, Gly^50^ and Gly^52^ Cα are significantly displaced by distances of 3.28 Å and 1.85 Å respectively, compared to hCK2 (PDB code: 2PVR) (Fig. 6).

### Differences between P*f*CK2α_D179S_ and hCK2

Six amino-acid substitutions are observed at the ATP binding site between the human (Arg^43^, Leu^45^, Glu^55^, Val^66^, His^115^, Val^116^) and *Plasmodium* enzyme (Lys^47^, Ile^49^, Asn^59^, Ala^70^, Tyr^119^, Ile^120^), giving rise to subtle alterations of the shape of the ATP binding pocket (Figs 3 and 5). As seen above, additional changes of the ATP binding pocket derive from a movement of strand β1. While C-lobe residues contributing to the ATP active site do not show significant variations compared to the active form of the human enzyme (PDB code: 2PVR), several changes arise from the N-lobe, with the Cα atoms of Gly^50^, Ile^49^ and Ala^70^ (located in the first and third strands of the N-Lobe) the most displaced. The main chain carbonyl atom of Ile^49^ comes to hydrogen bond distance with the O2 hydroxyl group of the ATP ribose (Fig. 5D). Likewise, the side chain CG1 atom of Val^57^ moves by 1.2 Å closer to the C4 of the base compared to hCK2. The aromatic side-chaisn of Tyr^119^ and Ile^120^ create a more hydrophobic interface compared to the human enzyme. Togetehr with Val^57^ and Ala^70^ they represent hydrophobic residues substitutions affecting the interaction with the adenine ring. In both cases Cα displacement (1.88 Å and 2.68 Å for Ala^70^ and Val^57^ respectively) allow the CG1 atom of Val57 to interact with the N9 amine of ATP. How substitutions of ATP binding residues in hCK2 significantly alter enzyme specificity was reported previously^36^ and thus, the alterations found in the *Pf*CK2 ATP binding pocket described here and at other locations at the surface of the enzyme (Fig. 6) might be relevant for designing specific inhibitors of the *Plasmodium* enzyme.

## Methods

### Cloning, expression and purification of *Pf*CK2α and its mutants

The cDNA of *Pf*CK2α was cloned into the pNIC28-Bsa4 vector using ligation-independent cloning as described^37^. In order to obtain a soluble protein suitable for structure determination, we used various N- and C-terminal truncations of the protein and placed the hexa-Histidine tag at either end of the polypeptide chain (Fig. 1A). Protein expression was conducted using BL21(DE3) Rosetta T1R cells with kanamycin and chloramphenicol at 37 °C inoculating 1 liter of Terrific broth with a 2% (v/v) *inoculum* until an OD_600_ of 0.6–0.8. The temperature was decreased to 16 °C. After 30 minutes, Isopropyl β-D-1-thiogalactopyranoside (Affymetrix) was added to the culture to a final concentration of 0.5 mM. After overnight incubation, cells were harvested at 4000 × g for 10 minutes and re-suspended in the lysis buffer (100 mM Na Hepes, 500 mM NaCl, 10 mM Imidazole, 10% (v/v) Glycerol and 0.5 mM Tris (2-carboxyethyl) phosphine (TCEP) at pH 8.0. Pellets stored at −80 °C were thawed in an iced-water bath with 25 µl of EDTA-free protease inhibitor cocktail (Calbiochem) and 10 mg lysozyme added to the thawed cell re-suspension. Sonication was applied in cycles of 3 seconds pulses followed by 5 seconds delays at 30% amplitude for 5 minutes (Sonics Vibra-cell). Cells debris were discarded following centrifugation at 47.000 × g for 30 minutes at 4 °C. The supernatant was filtered with a 1.2 µm pore syringe filter and subsequently loaded onto a 1 ml Ni-NTA Superflow column (Qiagen). Proteins were eluted using a stepwise gradient with 8%, 22% and 100% of a 500 mM imidazole solution in a buffer containing 20 mM Na Hepes, 500 mM NaCl, 10% (v/v) Glycerol and 0.5 mM TCEP at pH 7.5. Fractions eluting from the 22% imidazole buffer were pooled and concentrated by ultra-filtration with a 10 kDa cutoff concentrator (Vivascience) and loaded onto a preparative Hiload 16/60 Superdex 200prep column (GE) pre-equilibrated in 20 mM NaHepes, 300 mM NaCl, 10% (v/v) Glycerol and 0.5 mM TCEP at pH 7.5. Elution of *Pf*CK2α occurred as single and symmetric peak with an apparent molecular mass of 41 kDa. Protein identity was confirmed by SDS-PAGE (Fig. 1B) and mass spectrometry (39,665 Da). The protein was concentrated to 7 mg/ml, flash-frozen in liquid nitrogen and stored at −80 °C until use. The *Pf*CK2α_D179S_ mutant was generated using the QuickChange Protocol with forward primer GAAAATAGACAAATTAGATTAATTAGTTGGGGTCTAGCTGAATTTTATC, reverse primer GATAAAATTCAGCTAGACCCCAACTAATTAATCTAATTTGTCTATTTTC and template DNA vc026. Mutation was confirmed by sequencing. Expression and purification of the *Pf*CK2α_D179S_ mutant was performed following the same protocol as for the wild type enzyme. Using the same method, mutants *Pf*CK2α_Y30A,_ *Pf*CK2α_T63A_, *Pf*CK2α_T63AY30A_, *Pf*CK2α_Y30D_, *Pf*CK2α_T63D_, *Pf*CK2α_T63DY30D_ were obtained.

### Protein kinase autophosphorylation assay

A quantity of 2 µg of either wild type or D179S mutant of the *Pf*CK2α protein were incubated for 30 min at 30 °C in 20 µl of buffer containing 50 mM TrisHCl at pH 7.5, 50 mM MgCl_2_, [γ^32^P]-ATP and 8 µg of bovine serum albumin. The reaction was stopped by the addition of SDS sample buffer, boiled and the proteins resolved by SDS-PAGE. The gel was wrapped in a film and subjected to autoradiography with an X-ray film^38^.

### Protein kinase assay

Catalytic activity was quantified by measuring the incorporation of radiolabeled phosphate into a human CK2 substrate RRRDDDSDDD at a final concentration of 300 µM, as described earlier^39^. Human CK2 (CSNK2 DU813) served as positive control. One unit of activity was defined as the incorporation of 1 nanomole of radioactive phosphate into the substrate per minute. Specificity was calculated by dividing activity units by the amount in milligrams of assayed purified protein and substracting background level. Results were the averages of duplicate measurements.

### Inhibition activity assay

Two replicates were performed for hCK2 and *Pf*CK2, respectively. 2.5 μl of incremental concentrations of CX4945 compound was assayed with either enzyme in 50 μl total volume consisting of 50 mM Tris-HCl at pH 7.5, 10 mM DTT, 0.1 mM EGTA, 300 μM peptide substrate, 10 mM magnesium acetate and 100 μM γ^33^P-ATP. The reaction was incubated at 30 °C for 10 mins. Reactions were stopped by spotting 40 μl out of the 50 μl assay mixture onto 1.5 cm × 1.5 cm square of Whatman P81 paper, which were washed in 75 mM phosphoric acid, followed by acetone, then air dried. 1 ml Microscint 0 was added to tubes containing dried filter papers before counting on a liquid scintillation counter.

### Crystallization

*Pf*CK2α_D179S_ at a concentration of 10 mg.ml^−1^ was screened for crystallization with commercial kits in presence of 1 mM ATP using a mosquito^®^ crystallization robot (TTP Labtech). Very thin needles were obtained immediately after setting up, in Morpheus A9 conditions: 0.06 M Divalents; 0.1 M buffer system 3 at pH 8.5, 50% (v/v) Precipitant Mix 1, with a protein:precipitant ratio of 2:1. To grow larger crystals, these thin needles were added into protein solution, right before mixing with the precipitant. Crystals suitable for diffraction obtained by microseeding in 0.2 M Lithium Sulfate, 0.1 M BIS-TRIS pH 5.5, 25% (w/v) Polyethylene glycol 3,350 at 20 °C are displayed in Fig. 1A. Prior to flash-freezing in liquid nitrogen, crystals were transferred into a cryoprotectant solution containing the precipitant solution supplemented with 30% (v/v) glycerol.

### Data collection, structure determination and refinement

Diffraction data were collected on the PX-III beamline at SLS (Paul Sherrer institute, Villigen, Switzerland) and integrated using XDS^40^. Solvent content analysis using CCP4^24^ indicated the presence of three monomers in the asymmetric unit. The structure was determined using the molecular replacement program PHASER^24^ with the hCK2 structure (PDB code: 3NSZ) as a probe. Manual building using COOT^41^ was combined with refinement using BUSTER-2.10^42^ with tight NCS restraints between the three molecules related by ncs. Images of the structure were generated using PyMOL (http://www.pymol.org).

### Microcalorimetry

Isothermal titration microcalorimetry experiments were performed at 25 °C with a PEAQ-ITC isothermal titration calorimeter (Malvern). Protein concentration in the microcalorimeter cell (0.2 mL) was 50 μM. A total of 19 injections of 2 μl of ATP at a concentration of 500 μM were performed at intervals of 180 s while stirring at 600 rpm. The experimental data were fitted to theoretical titration curves using the manufacturer’s software.

### Mass spectrometry analysis

LC-MS/MS was done as previously described with minor modifications^43^. Briefly, *Pf*CK2 wild type and mutant protein gel bands were excised, reduced with DDT and alkylated with IAA, and then digested with trypsin. Tryptic peptides were separated and analyzed on a Dionex Ultimate 3000 RSLCnano system coupled to a Q-Exactive mass spectrometer (Thermo Electron, San Jose, USA) using a 60 min gradient. A full MS scan (350–1600 m/z range) was acquired at a resolution of 70,000 at m/z 200 and a maximum ion accumulation time of 100 ms. Dynamic exclusion was set as 30 s. Resolution for HCD spectra was set to 17,500 at m/z 200. The AGC setting of full MS scan and MS^2^ were set as 10^6^ and 2 × 10^5^, respectively. The ten most intense ions above a 1, 000 counts threshold were selected for HCD fragmentation with a maximum ion accumulation time of 100 ms. Isolation width of 2 Th was used for MS^2^. Single and unassigned charged ions were excluded from MS/MS. For HCD, normalized collision energy was set to 28%. The underfill ratio was defined as 0.1%. The MS raw file was converted into mgf format using ProteomeDiscoverer version 1.4. Protein identification was performed using Mascot server (version 2.4.1, Matrix Science, Boston, MA) against a *Plasmodium falciparum* protein database including the WT and MT *Pf*CK2α proteins (containing 5,649 sequence and 431,5306 residues). Mascot search was limited to a maximum of two missed trypsin cleavages, #13 C of 2, mass tolerance of 5 ppm for peptide precursors, and 0.02 Da mass tolerance for fragment ions. Fixed modification was carbamidomethyl at Cys residues, while variable modifications included oxidation at methionine residues, deamidation at asparagine and glutamine, and phosphorylation at serine, threonine and tyrosine. The extents of phosphorylation of detected phosphorylation sites were determined by integrating the areas of the extracted ion chromatograms of the unphosphorylated and phosphorylated peptides. The extracted ion chromatograms were extracted from a windows of +/−5 ppm of the precursor ions (see supplementary data).

### Data availability statement

Protein Data Bank accession code: The atomic coordinates and structure factors have been deposited in the Protein Data Bank with accession code 5XVU.

## Electronic supplementary material

supplementary information
