# Supplementary material for: The protein kinase CK2 catalytic domain from Plasmodium falciparum: crystal structure, tyrosine kinase activity and inhibition
Source: Sci Rep. 2018 May 9;8:7365. doi: 10.1038/s41598-018-25738-5 (PMC5943518; doi:10.1038/s41598-018-25738-5)
Supplement: Supplementary file 1 — supplementary information [file 41598_2018_25738_MOESM1_ESM.pdf]

The protein kinase CK2 catalytic domain from *Plasmodium falciparum*: crystal structure, tyrosine kinase activity and inhibition

Supplementary data (4 supplementary figures)

David Ruiz-Carrillo[1,2,&], Jianqing Lin[1,&], Abbas El Sahili[1,&], Meng Wei[1], Sze Siu Kwan[1], Peter CF Cheung[1], Christian Doerig[3], Julien Lescar[1,4*]

Legends for the supplementary Figures

Figure S1 The PfCK2 assymetric unit. A. View of PfCK2 asymmetric unit with monomers A, B and C shown as ribbons and colored in green cyan and pink respectively. The crystal lattice is built up such that the N-lobe of a *Pf*CK2α_{D179S} monomer interacts with the interdomain region that harbours the active site of a neighbouring molecule with the loop Tyr^{61}-Ans^{66} projecting towards the ATP binding site. The alpha helical C-lobe interacts back-to-back with neighbouring molecule C domain mediated by the N- terminal polypeptide chain (Ile^{12}-Ans^{38}) that is sandwiched between two molecules.

B. **Cartoon representation of the *Pf*CK2 monomer**. The ATP molecule occupying its active site is shown with sticks, overlaid with the volumes of the R- and C-spines shown in mesh representation colored in blue and brown, with the residues involved labeled.

Figure S2 Detailed MS analysis of the PfCK2 autophosphorylation. The table shows the various peptides identified.

Figure S3. Extracted ion chromatograms (XIC) of the detected un-modified and phosphorylated peptides were extracted with a window of +/- 5ppm of the precursor ions. The area under the XIC was used to determine the extents of phosphorylation sites of Y30 and T63. **A.** Upper panel showed the XIC of the unmodified peptide YSEVFNGYDTECNRPCAIK (3+); middle panel showed the phosphorylated peptide at T63; lower panel showed the two XICs plotted in the same scale. **B.** Upper panel showed the XIC of the unmodified peptide FYADVNIHKPKEYYDYDNLELQWNKPNRYEIMK (5+); middle panel showed the phosphorylated peptide at Y30 FYADVNIHKPKEYYDY#DNLELQWNKPNRYEIMK; lower panel showed the two XICs plotted in the same graph, the XIC of the phosphorylated peptides is at 100 x scale. **C.** Upper panel showed the XIC of the deamidated peptide FYADVNIHKPKEYYDYDN#LELQWNKPNRYEIMK (5+); middle panel showed the

deamidated and phosphorylated peptide at Y30 FYADVNIHKPKEYYDY#DN#LELQWNKPNRYEIMK; lower panel showed the two XICs plotted in the same graph, the XIC of the phosphorylated peptides is in 100X scale.

Figure S4 Isothermal Calorimetry measurement of the binding of *Pf*CK2α_{D179S} and ATP by ITC at 25°C. The syringe contained 500 μM ATP solution, and the cell contained 50 μM *Pf*CK2α_{D179S} protein.

A

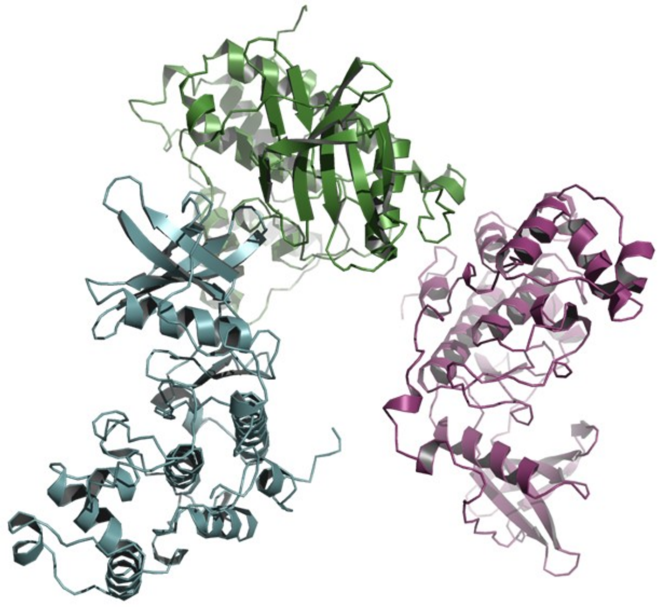

B

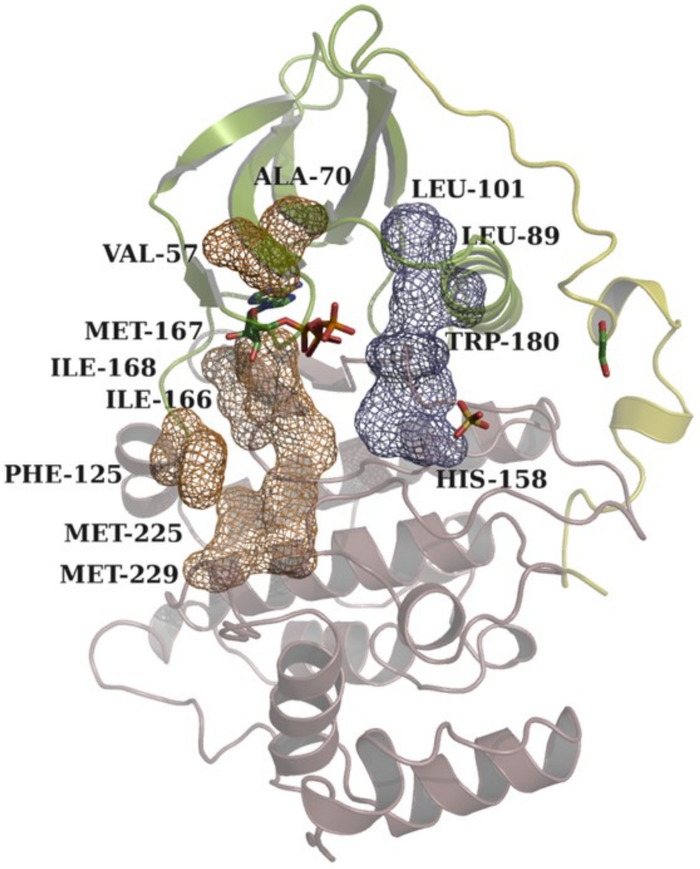
ALA-70
LEU-101
LEU-89
VAL-57
MET-167
TRP-180
ILE-168
ILE-166
PHE-125
HIS-158
MET-225
MET-229

Figure S1

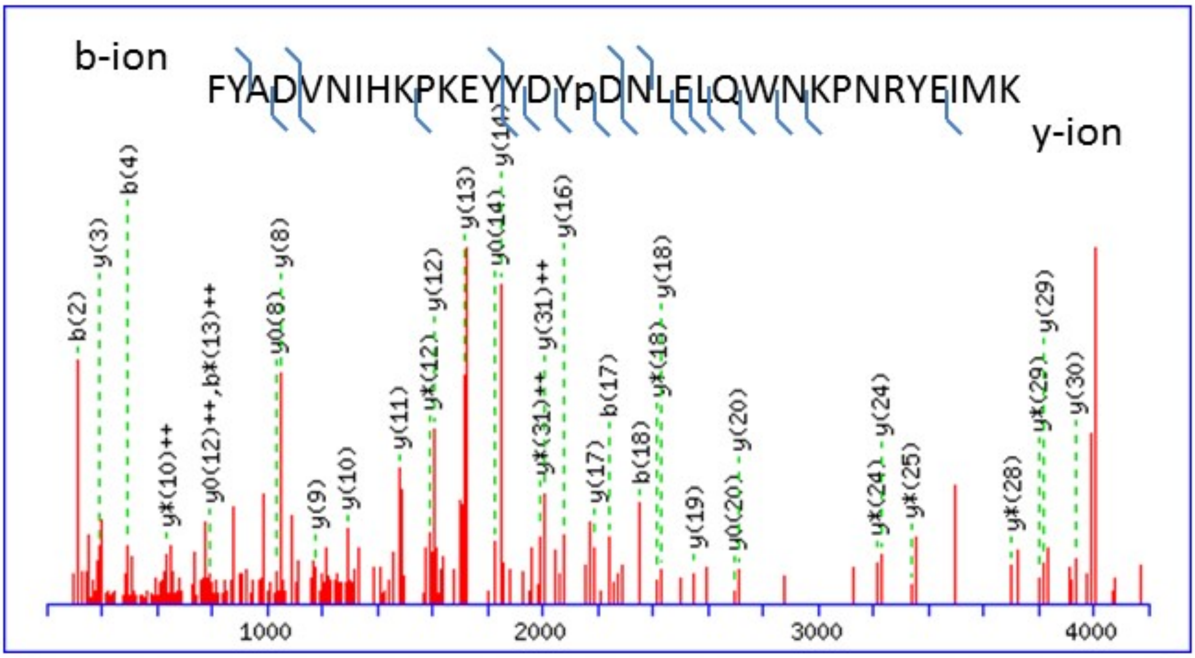

#	b	b^{++}	b^{*}	b^{*++}	b^{0}	b^{0++}	Seq.	y	y^{++}	y^{*}	y^{*++}	y^{0}	y^{0++}	#
1	148.0757	74.5415					F							33
2	311.1390	156.0731					Y	4169.9467	2085.4770	4152.9201	2076.9637	4151.9361	2076.4717	32
3	382.1761	191.5917					A	4006.8834	2003.9453	3989.8568	1995.4320	3988.8728	1994.9400	31
4	497.2031	249.1052			479.1925	240.0999	D	3935.8463	1968.4268	3918.8197	1959.9135	3917.8357	1959.4215	30
5	596.2715	298.6394			578.2609	289.6341	V	3820.8193	1910.9133	3803.7928	1902.4000	3802.8087	1901.9080	29
6	710.3144	355.6608	693.2879	347.1476	692.3039	346.6556	N	3721.7509	1861.3791	3704.7243	1852.8658	3703.7403	1852.3738	28
7	823.3985	412.2029	806.3719	403.6896	805.3879	403.1976	I	3607.7080	1804.3576	3590.6814	1795.8443	3589.6974	1795.3523	27
8	960.4574	480.7323	943.4308	472.2191	942.4468	471.7271	H	3494.6239	1747.8156	3477.5974	1739.3023	3476.6133	1738.8103	26
9	1088.5524	544.7798	1071.5258	536.2665	1070.5418	535.7745	K	3357.5650	1679.2861	3340.5384	1670.7729	3339.5544	1670.2809	25
10	1185.6051	593.3062	1168.5786	584.7929	1167.5946	584.3009	P	3229.4700	1615.2387	3212.4435	1606.7254	3211.4595	1606.2334	24
11	1313.7001	657.3537	1296.6735	648.8404	1295.6895	648.3484	K	3132.4173	1566.7123	3115.3907	1558.1990	3114.4067	1557.7070	23
12	1442.7427	721.8750	1425.7161	713.3617	1424.7321	712.8697	E	3004.3223	1502.6648	2987.2958	1494.1515	2986.3117	1493.6595	22
13	1605.8060	803.4066	1588.7795	794.8934	1587.7954	794.4014	Y	2875.2797	1438.1435	2858.2532	1429.6302	2857.2691	1429.1382	21
14	1768.8693	884.9383	1751.8428	876.4250	1750.8588	875.9330	Y	2712.2164	1356.6118	2695.1898	1348.0986	2694.2058	1347.6065	20
15	1883.8963	942.4518	1866.8697	933.9385	1865.8857	933.4465	D	2549.1531	1275.0802	2532.1265	1266.5669	2531.1425	1266.0749	19
16	2126.9259	1063.9666	2109.8994	1055.4533	2108.9154	1054.9613	Y	2434.1261	1217.5667	2417.0996	1209.0534	2416.1155	1208.5614	18
17	2241.9529	1121.4801	2224.9263	1112.9668	2223.9423	1112.4748	D	2191.0964	1096.0519	2174.0699	1087.5386	2173.0859	1087.0466	17
18	2355.9958	1178.5015	2338.9693	1169.9883	2337.9852	1169.4963	N	2076.0695	1038.5384	2059.0430	1030.0251	2058.0589	1029.5331	16
19	2469.0799	1235.0436	2452.0533	1226.5303	2451.0693	1226.0383	L	1962.0266	981.5169	1945.0000	973.0037	1944.0160	972.5116	15
20	2598.1225	1299.5649	2581.0959	1291.0516	2580.1119	1290.5596	E	1848.9425	924.9749	1831.9160	916.4616	1830.9320	915.9696	14
21	2711.2065	1356.1069	2694.1800	1347.5936	2693.1960	1347.1016	L	1719.8999	860.4536	1702.8734	851.9403	1701.8894	851.4483	13
22	2839.2651	1420.1362	2822.2386	1411.6229	2821.2545	1411.1309	Q	1606.8159	803.9116	1589.7893	795.3983	1588.8053	794.9063	12
23	3025.3444	1513.1758	3008.3179	1504.6626	3007.3339	1504.1706	W	1478.7573	739.8823	1461.7307	731.3690	1460.7467	730.8770	11
24	3139.3873	1570.1973	3122.3608	1561.6840	3121.3768	1561.1920	N	1292.6780	646.8426	1275.6514	638.3293	1274.6674	637.8373	10
25	3267.4823	1634.2448	3250.4558	1625.7315	3249.4717	1625.2395	K	1178.6350	589.8212	1161.6085	581.3079	1160.6245	580.8159	9
26	3364.5351	1682.7712	3347.5085	1674.2579	3346.5245	1673.7659	P	1050.5401	525.7737	1033.5135	517.2604	1032.5295	516.7684	8
27	3478.5780	1739.7926	3461.5515	1731.2794	3460.5674	1730.7874	N	953.4873	477.2473	936.4608	468.7340	935.4767	468.2420	7
28	3634.6791	1817.8432	3617.6526	1809.3299	3616.6685	1808.8379	R	839.4444	420.2258	822.4178	411.7126	821.4338	411.2205	6
29	3797.7424	1899.3749	3780.7159	1890.8616	3779.7319	1890.3696	Y	683.3433	342.1753	666.3167	333.6620	665.3327	333.1700	5
30	3926.7850	1963.8962	3909.7585	1955.3829	3908.7745	1954.8909	E	520.2799	260.6436	503.2534	252.1303	502.2694	251.6383	4
31	4039.8691	2020.4382	4022.8425	2011.9249	4021.8585	2011.4329	I	391.2374	196.1223	374.2108	187.6090			3
32	4170.9096	2085.9584	4153.8830	2077.4452	4152.8990	2076.9531	M	278.1533	139.5803	261.1267	131.0670			2
33							K	147.1128	74.0600	130.0863	65.5468			1

Figure S2

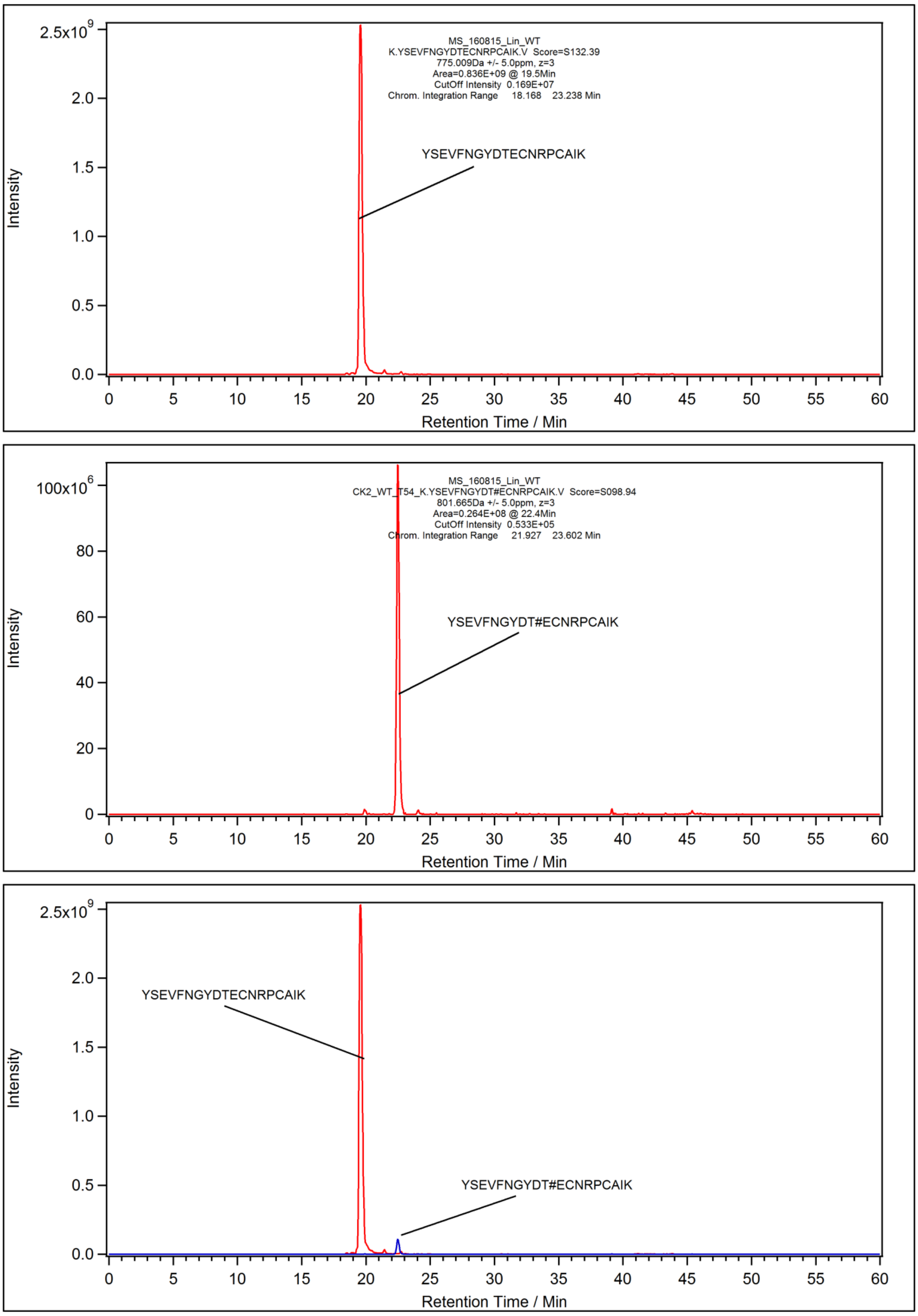
MS_160815_Lin_WT
K.YSEVFNGYDTECNRPCAIK.V Score=S132.39
775.009Da +/- 5.0ppm, z=3
Area=0.836E+09 @ 19.5Min
CutOff Intensity 0.169E+07
Chrom. Integration Range 18.168 23.238 Min
YSEVFNGYDTECNRPCAIK
Intensity
2.5x10^9
2.0
1.5
1.0
0.5
0.0
0 5 10 15 20 25 30 35 40 45 50 55 60
Retention Time / Min
MS_160815_Lin_WT
CK2_WT_T54_K.YSEVFNGYDT#ECNRPCAIK.V Score=S098.94
801.665Da +/- 5.0ppm, z=3
Area=0.264E+08 @ 22.4Min
CutOff Intensity 0.533E+05
Chrom. Integration Range 21.927 23.602 Min
YSEVFNGYDT#ECNRPCAIK
Intensity
100x10^6
80
60
40
20
0
0 5 10 15 20 25 30 35 40 45 50 55 60
Retention Time / Min
YSEVFNGYDTECNRPCAIK
YSEVFNGYDT#ECNRPCAIK
Intensity
2.5x10^9
2.0
1.5
1.0
0.5
0.0
0 5 10 15 20 25 30 35 40 45 50 55 60
Retention Time / Min

Figure S3A

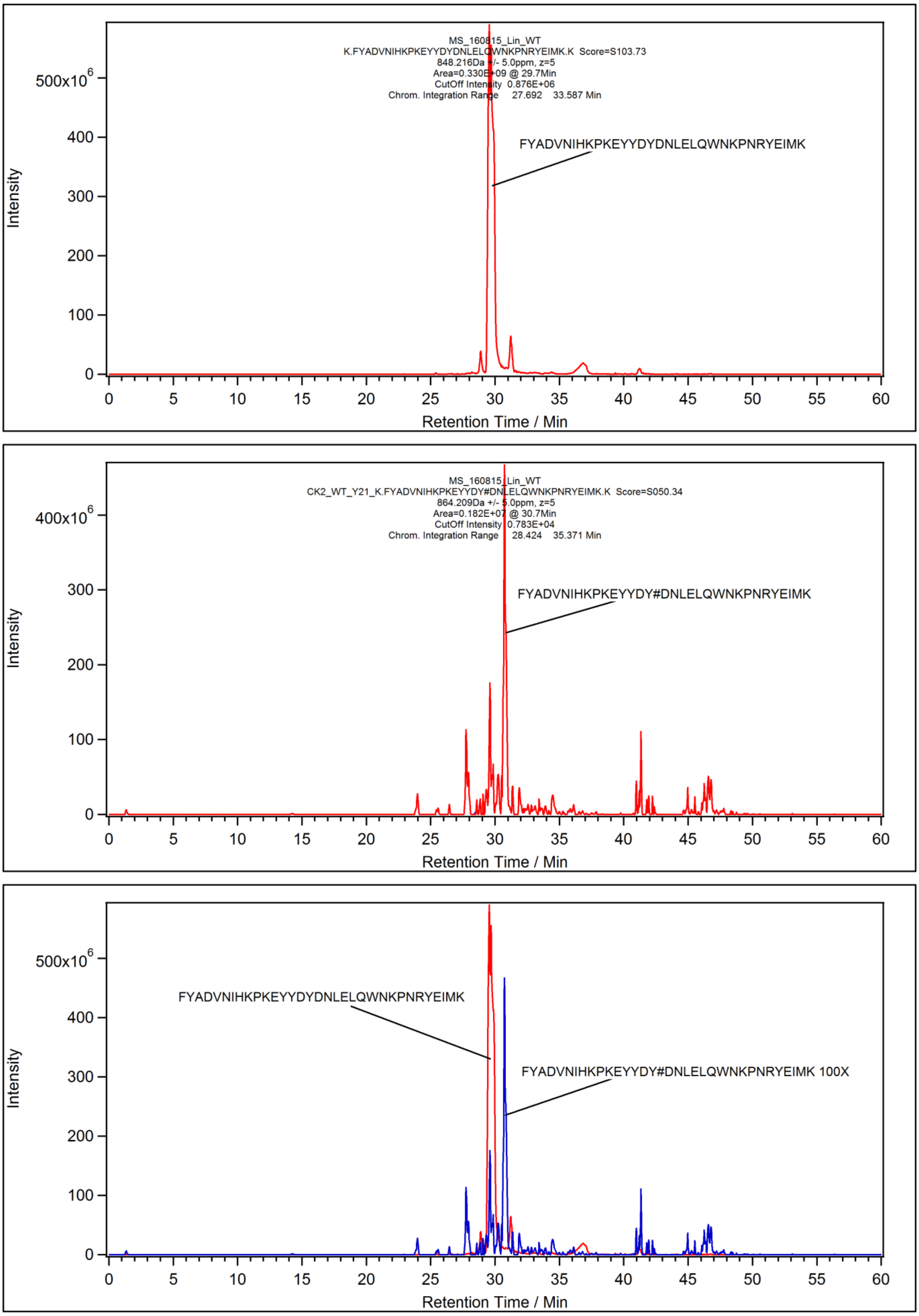

MS_160815_Lin_WT
K.FYADVNIHKPKEYYDYDNLELQWNKPNRYEIMK.K Score=S103.73
848.216Da +/- 5.0ppm, z=5
Area=0.330E+09 @ 29.7Min
CutOff Intensity 0.876E+06
Chrom. Integration Range 27.692 33.587 Min
FYADVNIHKPKEYYDYDNLELQWNKPNRYEIMK
Intensity
500x10^6
400
300
200
100
0
0 5 10 15 20 25 30 35 40 45 50 55 60
Retention Time / Min
MS_160815_Lin_WT
CK2_WT_Y21_K.FYADVNIHKPKEYYDY#DNLELQWNKPNRYEIMK.K Score=S050.34
864.209Da +/- 5.0ppm, z=5
Area=0.182E+07 @ 30.7Min
CutOff Intensity 0.783E+04
Chrom. Integration Range 28.424 35.371 Min
FYADVNIHKPKEYYDY#DNLELQWNKPNRYEIMK
Intensity
400x10^6
300
200
100
0
0 5 10 15 20 25 30 35 40 45 50 55 60
Retention Time / Min
FYADVNIHKPKEYYDYDNLELQWNKPNRYEIMK
FYADVNIHKPKEYYDY#DNLELQWNKPNRYEIMK 100X
Intensity
500x10^6
400
300
200
100
0
0 5 10 15 20 25 30 35 40 45 50 55 60
Retention Time / Min

Fig. S3B

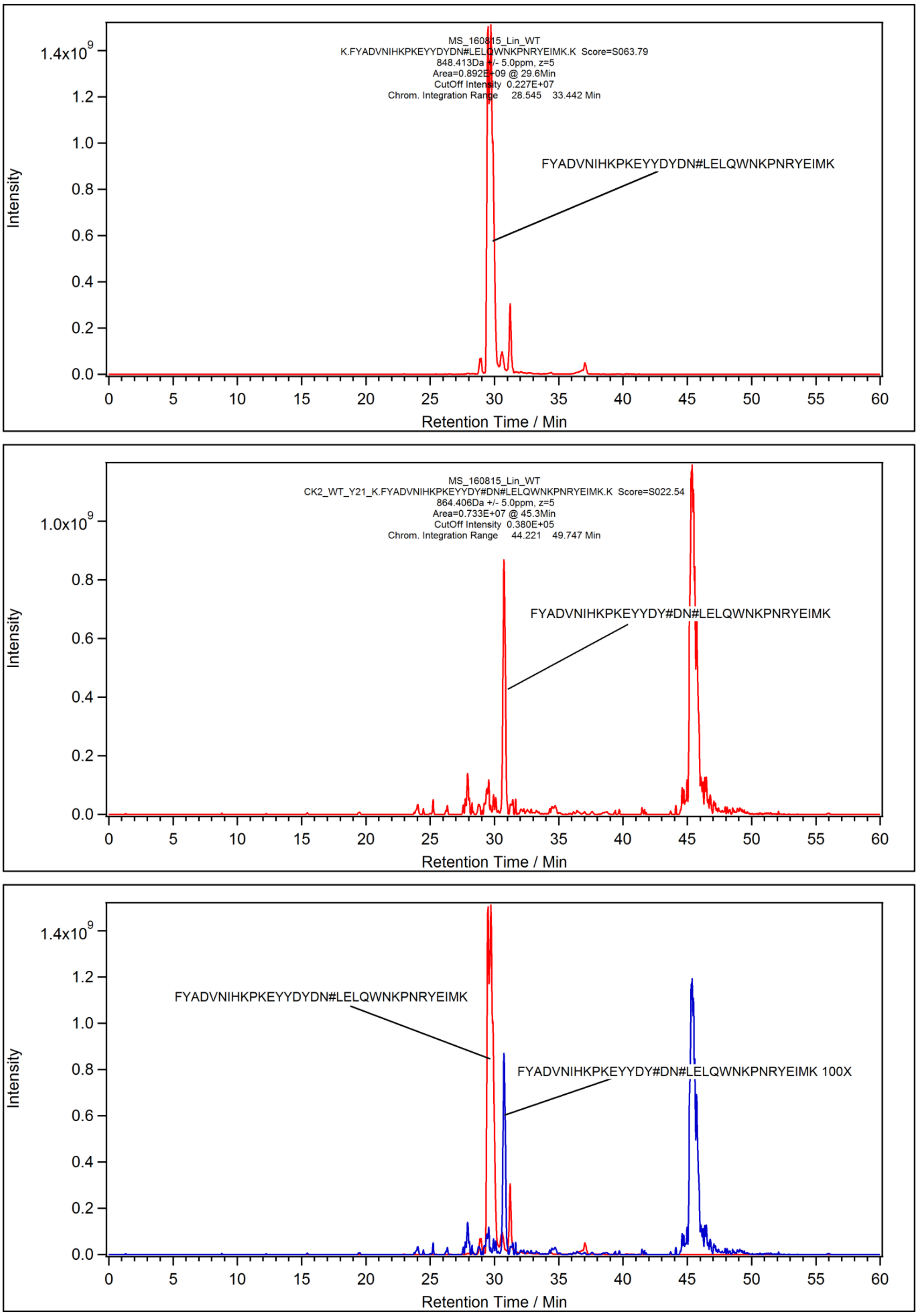
MS_160815_Lin_WT
K.FYADVNIHKPKEYYDYDN#LELQWNKPNRYEIMK.K Score=S063.79
848.413Da +/- 5.0ppm, z=5
Area=0.892E+09 @ 29.6Min
CutOff Intensity 0.227E+07
Chrom. Integration Range 28.545 33.442 Min
FYADVNIHKPKEYYDYDN#LELQWNKPNRYEIMK
Intensity
Retention Time / Min
MS_160815_Lin_WT
CK2_WT_Y21_K.FYADVNIHKPKEYYDY#DN#LELQWNKPNRYEIMK.K Score=S022.54
864.406Da +/- 5.0ppm, z=5
Area=0.733E+07 @ 45.3Min
CutOff Intensity 0.380E+05
Chrom. Integration Range 44.221 49.747 Min
FYADVNIHKPKEYYDY#DN#LELQWNKPNRYEIMK
Intensity
Retention Time / Min
FYADVNIHKPKEYYDYDN#LELQWNKPNRYEIMK
FYADVNIHKPKEYYDY#DN#LELQWNKPNRYEIMK 100X
Intensity
Retention Time / Min

Fig. S3C

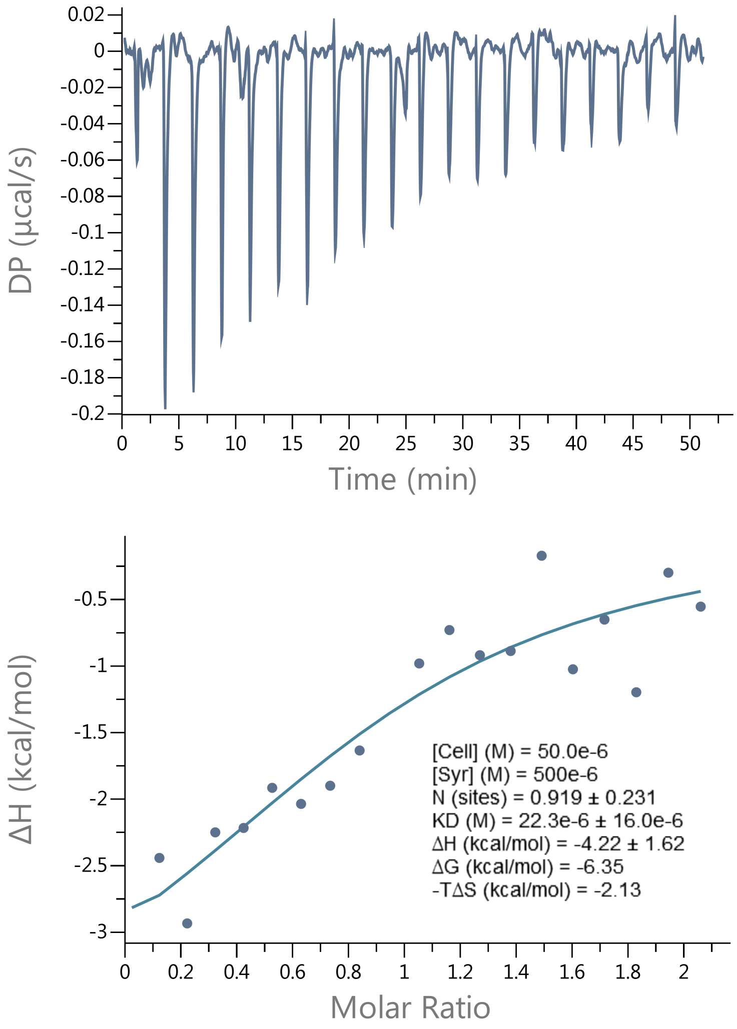
0.02
0
-0.02
-0.04
-0.06
-0.08
-0.1
-0.12
-0.14
-0.16
-0.18
-0.2
DP (µcal/s)
0
5
10
15
20
25
30
35
40
45
50
Time (min)
-0.5
-1
-1.5
-2
-2.5
-3
ΔH (kcal/mol)
[Cell] (M) = 50.0e-6
[Syr] (M) = 500e-6
N (sites) = 0.919 ± 0.231
KD (M) = 22.3e-6 ± 16.0e-6
ΔH (kcal/mol) = -4.22 ± 1.62
ΔG (kcal/mol) = -6.35
-TΔS (kcal/mol) = -2.13
0
0.2
0.4
0.6
0.8
1
1.2
1.4
1.6
1.8
2
Molar Ratio

Figure S4
